# Investigation of antimicrobial use at a tertiary care hospital in Southern Punjab, Pakistan using WHO methodology

**DOI:** 10.1186/s13756-017-0199-7

**Published:** 2017-04-28

**Authors:** Muhammad Atif, Muhammad Azeem, Anum Saqib, Shane Scahill

**Affiliations:** 1 Department of Pharmacy, The Islamia University of Bahawalpur, Bahawalpur, Pakistan; 2 School of Management, Massey University, Auckland, New Zealand

**Keywords:** Irrational prescribing, WHO, Antimicrobial utilization, Rational, Antimicrobial use indicators, Antimicrobial resistance

## Abstract

**Background:**

Globally, between 20 to 50% of antimicrobial consumption is inappropriate, causing significant impact on the quality of care, cost of therapy and incidence of adverse drug reactions. The purpose of this study was to investigate the prescribing patterns and utilization of antimicrobials in ten selected wards at Bahawal Victoria Hospital (BVH), Bahawalpur, Punjab, Pakistan.

**Methods:**

A descriptive cross-sectional study was designed using the World Health Organization (WHO) indicators for antimicrobial use. Standard data collection forms were used in ten wards and the Pharmacy Department at BVH. Antimicrobial utilization patterns in terms of frequency and percentage were also determined. Systematic random sampling techniques were used to collect data from 1,000 prescription records out of 21,115 prescriptions written for the six months January to June 2016.

**Results:**

For the hospital indicators, a formulary list or essential medicines list (FL/EML) was available, but standard treatment guidelines (STGs) for infectious diseases was not. The average number of days that key antimicrobials were out of stock was 3.3 days per month. The expenditure on antimicrobials as a percentage of the total medicines costs was 12.2%. For the prescribing indicators, the percentage of hospitalizations with antimicrobial(s) prescribed was 82.3%, and the average number of antimicrobials per hospitalization was 1.4 (SD = 0.6). The average duration of antimicrobial treatment per hospitalization was 5.4 days (SD = 3.2). The average cost of antimicrobials prescribed per hospitalization was USD 5.4 (SD = 6.7). None of the patients who were prescribed antimicrobials, received AM according to the STGs (pneumonia and cesarean section cases). Among the patient-care and supplemental indicators, the average duration of hospital stay of patients who received antimicrobials was 6.4 (SD = 4.3) days. The drug sensitivity testing was almost non-existent, with only 0.24% prescription records having drug sensitivity tests. Ceftriaxone (39.6%), metronidazole (23.4%) and cefotaxime (23.1%) were the top most frequently prescribed antimicrobials.

**Conclusions:**

The results of the current study revealed less than optimal antimicrobial prescribing and utilization patterns of selected wards at BVH. Continuous education and training of physicians, and cost-effective policies could play an important role in promoting the rational use of antimicrobials in this setting.

**Electronic supplementary material:**

The online version of this article (doi:10.1186/s13756-017-0199-7) contains supplementary material, which is available to authorized users.

## Background

There is no doubt that antimicrobials have played a revolutionary role in healthcare systems worldwide [1]. From the discovery of the very first antibiotics in the 1930s and 1940s, these medicines have saved countless lives [1, 2]. This has been through reducing morbidity and mortality rates for number of infectious diseases that were once a principal cause of death [3, 4]. However, the problem is not completely resolved as infectious diseases account for 20% of deaths globally; equating to 11 million deaths per annum [4]. This is largely due to the emergence of antimicrobial resistance (AMR), a phenomenon that was first observed in 1947, when microorganisms (*Staphylococcus* species) showed resistance against penicillin [5]. The rapidly emerging multi drug resistant (MDR) microbial species make the treatment options very limited, problematic, costly and with greater incidences of adverse drug reactions (ADRs) [2, 6].

A number of factors are thought to be associated with this rapidly developing AMR, but a large number of studies support the claim that inappropriate antimicrobial use (either prescribing antimicrobials when not required or prescribing a broad spectrum agent when a narrower spectrum agent would be adequate) [7, 8] is the main determinant of AMR [9–14]. One study has claimed that almost 30 to 50% of hospitalized patients receive at least one antimicrobial, and therefore, antimicrobials account for greater than 30% of total hospital budgets [15]. According to available literature, 20 to 50% of total antimicrobial consumption is inappropriate [13, 16, 17], causing significant impact on the quality of care [18], cost of therapy [19, 20] and incidence of ADRs [21].

Although, the problems associated with antimicrobials exist all over the world the developing countries are afflicted the most where infection rates are much higher and resources are very limited [22, 23]. For example, in Pakistan, due to limited resources, physicians working in government hospitals are forced to prescribe antimicrobials that have little or no effect against a number of microbes [24]. A number of studies from Pakistan have already reported high prescribing rates of antimicrobials as 51.5% [25], 52% [26], 52.4% [27], 48.9% [28]. However, very limited data is available from Pakistan [29] about the antimicrobial prescribing patterns relating to the World Health Organization (WHO) antimicrobial use indicators [22]. Based on aforementioned reasons, it is important to implement a continuous antimicrobial consumption surveillance system using standard methodology in hospitals as part of AMR prevention strategies [30]. For this purpose, the WHO has developed a set of indicators to measure the prescribing and use of antimicrobials in hospitals [22]. These indicators are classified as hospital, prescribing, patient-care, and supplemental indicators.

The purpose of this study was to investigate the use and prescribing patterns of antimicrobials at 10 wards in the Bahawal Victoria Hospital (BVH), Bahawalpur, Punjab, Pakistan. The findings of the current study could be used to benchmark policy and practice activities regarding quality of antimicrobial use. Specifically, this insight will help policy-makers to implement appropriate interventions designed to improve the judicious use of antimicrobials in Pakistan and more globally.

## Methods

### Study settings

The study was conducted in the BVH, Bahawalpur, Punjab, Pakistan. The BVH is a tertiary level 1600-bed hospital with all specialties. Around 350 physicians, 20 pharmacists, 400 nurses and 3,000 paramedical staff attend an average of 90,000 patients per month [27]. Purposively, ten wards and the Pharmacy Department of the hospital were selected to collect the required data. Each of the wards had at least one pharmacist. The characteristics of the selected wards are summarized in Table 1.

**Table 1:** Characteristics of the selected wards at the Bahawal Victoria Hospital

Sr. No.	Ward name	No. of medical doctors	Paramedical staff^a^	No of beds	Patient turn over (monthly)
1	Chest Disease Unit	25	23	60	295
2	Ear Nose Throat	16	15	42	176
3	Gynecology	20	29	75	346
4	Medical 1	31	31	80	729
5	Medical 2	35	28	75	753
6	Nephrology	7	10	40	63
7	Orthopedics	40	32	88	500
8	Surgical 4	27	22	70	309
9	Skin	21	12	20	48
10	Urology	28	38	90	300

^a^Paramedical staff includes nurses, ward boys and sweepers working in the ward

### Study design and outcome variables

It was a non-experimental and descriptive cross-sectional study, designed according to the study objectives. The outcome indicators are related to four general areas of antimicrobial use; hospital, prescribing, patient-care and supplemental indicators. The antimicrobial use patterns in terms of frequency and percentage of single as well as multiple antimicrobials were also determined. The Anatomical Therapeutic Chemical (ATC) classification system was used for the coding of antimicrobials. The defined daily doses (DDDs) of antimicrobials were calculated and their comparison across wards was also determined [31].

### Study inclusion/exclusion criteria

The inclusion/exclusion criteria are provided in Additional file 1: Table S1.

### Sampling and data collection

We used the standard indicator forms to collect data. Data reliability was ensured by following the WHO guidelines and methods [22, 32]. The data was collected during the months of June to July 2016.

#### Hospital indicators

The data regarding the hospital indicators were collected for a period of one year (i.e. July 2015 to June 2016), except for indicator 4 (the average number of days that a set of key antimicrobials is out of stock), that was determined based on the data collected for a duration of six months (i.e. January to June 2016). The most recent copies of the Formulary List or Essential Medicines List (FL/EML), key antimicrobials and standard treatment guidelines (STGs) were obtained from the Pharmacy Department. The WHO hospital indicators, their data sources and the standard data collection forms are in Additional file 1: Table S2.

**Table 2:** Demographic characteristics of the patients

Patient Variables		Results
		*n* (%)
Gender	Male	446 (44.6)
	Female	554 (55.4)
Age (years)	18–35	276 (27.6)
	36–55	199 (19.9)
	>55	525 (52.5)
Residence	Rural	740 (74)
	Urban	260 (26)
Income^a^ (Pakistani Rupees per annum)	<300,000	656 (65.6)
	300,000–1000,000	338 (33.8)
	>1000,000	6 (0.6)
Comorbidity	Present	554 (55.4)
	Absent	454 (45.4)

^a^1USD = 104.81PKR

#### Prescribing, patient-care and supplemental indicators, and prescribing patterns of antimicrobials

One thousand prescription records (100 per ward) out of a total of 21,115 prescriptions written from January to June 2016 were selected. To minimize selection bias prescription records written for each ward were divided into four parts, and from each part 25 prescriptions were selected using a systematic random sampling technique [33]. The sampling unit was the prescriptions records written for inpatients only. From these prescription records, prescribing indicators, patient-care indicators, supplemental indicator, and prescribing patterns of antimicrobials were determined. The WHO prescribing, patient-care and supplemental indicators, their data sources and standard data collection forms are summarized in Additional file 1: Table S3.

**Table 3:** WHO hospital indicators

Sr. No.	Parameter	Results
1	Existence of DTC	Yes
2	Existence of STGs for infectious diseases	No
3	Existence of FL/EML	Yes
4	Total number of antimicrobials on the FL/EML	25 generics
5	Are all medicines identified by INN	Yes
6	Availability of a set of key antimicrobials in the hospital stores on the day of study	93.8%
7	Average number of days that a set of key antimicrobials is out of stock	3.3 days/month
8	Total number of hospital discharges during the last calendar year	128,940
9	Surgical interventions performed during the last calendar year	Major = 28,257
		Minor = 17,739
10	Expenditure on antimicrobials^a^ as a percentage of the total hospital medicine costs	12.2%

*DTC* Drug and therapeutic committee, *STGs* Standard treatment guidelines, *FL/EML* Formulary list/essential medicines list, *INN* International non-proprietary names

^a^Annual bulk purchase data only

### Data analysis

Statistical Package for Social Sciences (IBM Corp. Released 2012. IBM SPSS Statistics for Windows, Version 21.0. Armonk, NY: IBM Corp.) and Microsoft Excel (MS Office 2010) were used for data analysis. Descriptive statistics were used to present the results. Difference in performance among various wards was established using the ANOVA test. Statistical significance was determined at *p* < 0.05. Conversion rate of United States Dollars (USD) to Pakistan Rupees (PKR) was 1USD = 104.81PKR.

## Results

### Demographic characteristics

From the 1,000 prescription records, 44.6% patients were male and 55.4% were female. The demographic characteristics of the patients included in the current study are summarized in Table 2.

### Hospital indicators

The Drug and Therapeutic Committee (DTC) functions in an ongoing basis in the BVH, and the hospital had a FL/EML that contains 25 generic antimicrobials. The results of the hospital indicators are summarized in Table 3.

Twenty five antimicrobials listed in FL/EML were available across 32 different dosage forms. Out of these 32 dosage forms, 30 (93.8%) were available (in stock) on the study day (Additional file 1: Table S4). The average number of days that a set of key antimicrobials was out of stock was 3.3 days per month (Additional file 1: Table S5).

**Table 4:** Prescribing indicators at the selected wards in the Bahawal Victoria Hospital (*n* = 1,000) (*Indicators 6–10, 13, 14*)

Hospital Wards	Prescribing indicators							
	Percent of hospitalizations with one or more AMs	Average AMs per hospitalization in which AMs were prescribed (SD)	Percent of AMs prescribed from FL/EML	Average cost (USD) of AMs prescribed per hospitalization in which AMs were prescribed (SD)	Average duration of prescribed AMs treatment days (SD)	Percent of pneumonia patients who received AMs	Percent of patients who received AMs for Pneumonia in accordance with clinical guidelines^a^	Percent of AMs prescribed by INN
1. CDU	91	1.2 (0.4)	100	4.6 (8.7)	5.4 (2.7)	14	0	41.6
2. ENT	89	1.3 (0.6)	100	3.2 (2.3)	5 (2.7)	0	-	78.6
3. Gynecology	91	2 (0)	100	9.5 (5.6)	6 (3.6)	0	-	50
4. Medical 1	89	1.3 (0.6)	98.9	5.8 (6.2)	5 (3.4)	8	0	20.5
5. Medical 2	91	1.3 (0.5)	100	6.7 (9.4)	5.4 (3.8)	6	0	18.5
6. Nephrology	63	1.3 (0.5)	100	7.2 (10.2)	7 (2.1)	0	-	62.7
7. Orthopedics	65	1.5 (0.7)	100	3.4 (3.5)	2.6 (1.3)	0	-	65.7
8. Surgical 4	95	1.4 (0.7)	100	6.9 (5.5)	5.8 (3.3)	0	-	8
9. Skin	63	1.3 (0.6)	100	2.4 (3.7)	5.5 (2.6)	0	-	12.1
10. Urology	86	1.3 (0.5)	100	3.6 (3.2)	6.3 (3.3)	0	-	55.6
Mean (SD)/Percentage	82.3	1.4 (0.6)	99.9	5.4 (6.7)	5.4 (3.2)	0.03	0	52.5
ANOVA	-----	*p* < 0.005^1^	-----	*p* < 0.005^1^	*p* < 0.005^1^	------	------	-----

*AMs* Antimicrobials, *FL/EML* Formulary list/essential medicines list, *USD* United States Dollars, *INN* International non-proprietary name, *CDU* Chest Disease Unit, *ENT* Ear Nose Throat

^a^Infectious Diseases Society of America/American Thoracic Society Consensus Guidelines on the Management of Community-Acquired Pneumonia in Adults (https://www.thoracic.org/statements/resources/mtpi/idsaats-cap.pdf)

^1^Bonferroni correction results are included in Additional file 2

**Table 5:** Surgical antimicrobial prophylaxis indicators (Indicators 11, 12)

Sr. No.	Parameters	Results
1	Number of cesarean section cases	100
2	Surgical antimicrobial prophylaxis prescribed for patients	54
3	Total number of doses of surgical antimicrobial prophylaxis prescribed for cesarean section procedures	65
4	Average number of doses of surgical antimicrobial prophylaxis prescribed for cesarean section procedures	1.2 ± 0.4
5	Percentage of patients who received surgical antimicrobial prophylaxis for cesarean section in accordance with clinical guidelines^a^	0

^a^Clinical Practice Guidelines for Antimicrobial Prophylaxis in Surgery (www.ashp.org/surgical-guidelines)

In the BVH, for the year 2015–2016 approximately USD 6.58 million was allocated for the purchase of medicines. The expenditure on antimicrobials as a percentage of the total spend was 12.2% (USD 0.8 million). The proportion costs of individual antimicrobial was also calculated (Additional file 1: Table S6).

**Table 6:** Patient-care and supplemental indicators at the selected wards in the Bahawal Victoria Hospital (*Indicators 15–17*)

Hospital Wards	Patient-care and supplemental indicators			
	Number of AM doses prescribed Sum (mean ± SD)	Percent of doses of prescribed AM actually administered	Average duration of hospital stay of patients who received AM (SD)	Number of AM drug sensitivity tests reported per hospital admission with curative AM prescribed
1. CDU	1134 (12.5 ± 7.7)	100	5.8 (3.2)	1
2. ENT	1116 (12.5 ± 7.8)	100	5.1 (2.8)	0
3. Gynecology	2635 (29 ± 17.1)	100	6.3 (4.1)	0
4. Medical 1	1188 (13.4 ± 11.4)	100	5.9 (3.9)	1
5. Medical 2	1231 (13.5 ± 10.2)	100	5.9 (3.9)	0
6. Nephrology	1282 (20.4 ± 11.9)	100	8.2 (2.8)	0
7. Orthopedics	452 (7 ± 5.3)	100	3.5 (2.5)	0
8. Surgical 4	1663 (17.5 ± 11.3)	100	7.3 (5.8)	0
9. Skin	1049 (16.6 ± 8.2)	100	7.5 (5.3)	0
10. Urology	1297 (15.1 ± 9.3)	100	8.2 (5.1)	0
Mean (SD)/ Percentage	13047 (15.9 ± 12)	100	6.4 (4.3)	0.002%
ANOVA	*p* < 0.005^*^	-----	*p* < 0.005	-----

*AM* Antimicrobial, *CDU* Chest Disease Unit, *ENT* Ear Nose Throat; ^*^It is not a standard indicator, but is mandatory to calculate the indicator 15

### Prescribing indicators

The percentage of hospitalizations with antimicrobial(s) prescribed was 82.3%, and from these patients, the average number of antimicrobials per hospitalization was 1.4 (SD = 0.6). The results regarding prescribing indicators in the selected wards are summarized in Table 4.

From a total of 100 cesarean section cases, none received surgical antimicrobial prophylaxis in accordance with the clinical guidelines (Table 5).

### Patient-care and supplemental indicators

The results regarding prescribing indicators are summarized in Table 6.

Antimicrobial utilization was determined based on the WHO’s DDDs recommendations [31]. The total antimicrobial consumption in the selected wards varied between 2.32 DDD/1000 hospitalization days and 322.66 DDD/1000 hospitalization days. It is worth noting that 1.2 g amoxiclav had the highest consumption, in the range 0.5 DDD/1000 hospitalization days to 209 DDD/1000 hospitalization days (Table 7).

**Table 7:** DDDs/1000 hospitalization days of the antimicrobials being prescribed at the BVH and their comparison across the selected wards

Sr. No.	Antimicrobial	Route	WHO DDD	CDU	ENT	Gynecology	M 1	M 2	Nephrology	Orthopedics	S4	Skin	Urology	Total	Mean
1	Amikacin 0.5 g	Parenteral	1 g	---	---	---	---	---	---	1.92	---	---	2.38	4.3	2.15 g
2	Ampicillin 0.5 g	Parenteral	2 g	---	10.51	---	---	---	---	---	---	---	---	10.51	10.51 g
3	Amoxiclav 0.625 g	Oral	1 g	2.48	2.60	---	0.85	2.40	22.20	---	8.13	81.28	---	119.94	17.13 g
4	Amoxiclav 1.2 g	Parenteral	3 g	0.5	3.63	---	6.52	3.39	89.8	---	9.82	209.0	---	322.66	46.09 g
5	Cefoperazone + Sulbactam 1 g	Parenteral	4 g	---	---	---	---	---	---	3.33	---	---	16.13	19.46	9.73 g
6	Cefotaxime 0.25 g	Parenteral	4 g	0.28	2.62	1.62	---	---	1.74	---	0.03	---	0.32	6.61	1.1 g
7	Cefotaxime 1 g	Parenteral	4 g	1.93	17.42	16.61	---	---	20.6	---	0.32	---	2.68	59.56	9.92 g
8	Ceftriaxone 0.25 g	Parenteral	2 g	3.11	0.23	---	1.64	0.99	---	3.10	3.18	2.68	1.27	16.2	2.02 g
9	Ceftriaxone 1 g	Parenteral	2 g	18.4	7.38	---	9.78	11.84	---	14.61	28.27	37.24	5.82	133.34	16.7 g
10	Ciprofloxacin 0.4 g	Parenteral	0.5 g	9.37	14.54	---	3.2	2.36	45.95	3.89	1.63	56.56	30.19	167.69	18.6 g
11	Ciprofloxacin 0.5 g	Oral	1 g	1.67	3.40	---	0.73	0.57	18.14	1.02	0.37	22.72	12.78	61.4	6.82 g
12	Clarithromycin 0.25 g	Oral	0.5 g	3.55	---	---	---	0.16	---	---	---	---	---	3.71	1.85 g
13	Clarithromycin 0.5 g	Oral	0.5 g	2.30	---	---	0.02	---	---	---	---	---	---	2.32	1.16 g
14	Clarithromycin 0.5 g	Parenteral	1 g	2.92	---	---	0.13	0.04	---	---	---	---	---	3.09	1.03 g
15	Gentamicin 0.12 g	Parenteral	0.24 g	---	2.74	---	---	---	---	---	1.67	---	---	4.41	2.2 g
16	Metronidazole 0.4 g	Oral	2 g	1.21	1.13	4.43	0.5	0.47	2.56	0.20	2.49	4.29	0.1	17.38	1.73 g
17	Metronidazole 1 g	Parenteral	1.5 g	2.65	6.56	44.74	4.7	2.95	13.60	1.87	14.13	15.15	1.05	107.4	10.74 g
18	Moxifloxacin0.4 g	Parenteral	0.4 g	2.30	---	---	0.82	0.24	33.25	---	---	---	1.05	37.66	7.53 g
19	Moxifloxacin 0.4 g	Oral	0.4 g	1.88	---	---	0.54	---	18.14	---	---	---	0.53	21.09	5.28 g
20	Vancomycin 0.5 g	Parenteral	2 g	---	---	---	1.05	1.35	7.93	---	---	---	---	10.33	3.5 g

*WHO* World Health Organization, *DDD* Defined daily dose, *CDU* Chest Disease Unit, *ENT* Ear Nose Throat, *M1* Medical 1, *M2* Medical 2, *S4* Surgical 4

### Prescribing patterns of antimicrobials

Out of 823 (82.3%) prescriptions with antimicrobial(s) prescribed, 536 (65.13%) had one antimicrobial, 252 (30.62%) included two antimicrobials and 34 (4.13%) had three antimicrobials. Ceftriaxone (39.61%), metronidazole (23.45%) and cefotaxime (23.09%) were the most frequently prescribed antimicrobials in the selected wards (Table 8). Ceftriaxone was the most commonly prescribed antimicrobial in the Chest Disease Unit (CDU), Medical 1, Medical 2, Orthopedics and Surgical 4 wards (Additional file 1: Table S7).

**Table 8:** Frequency of various antimicrobials being prescribed at the selected wards of the Bahawal Victoria Hospital (*n* = 823)

Sr. No.	Antimicrobial name	ATC Code	No. of hospitalizations	Percentage
1	Ceftriaxone	J01DD04	326	39.6
2	Metronidazole	J01XD01	193	23.4
3	Cefotaxime	J01DD01	190	23.1
4	Amoxiclav	J01CR02	162	19.7
5	Ciprofloxacin	J01MA02	110	13.4
6	Cefoperazone	J01DD12	62	7.5
7	Clarithromycin	J01FA09	22	2.7
8	Moxifloxacin	J01MA14	20	2.4
9	Cephradine	J01DB09	16	1.9
10	Vancomycin	J01XA01	12	1.5
11	Ampicillin	J01CA01	11	1.3
12	Gentamicin	J01GB03	11	1.3
13	Amikacin	J01GB06	8	1

*ATC* Anatomical therapeutic chemical classification system

The patients admitted to the selected wards of the BVH were also prescribed antimicrobial combinations. The most frequently prescribed combinations were cefotaxime with metronidazole (11.5%) and ceftriaxone with metronidazole (4.4%) (Table 9).

**Table 9:** Most commonly prescribed antimicrobial combinations at the selected wards of the Bahawal Victoria Hospital

Sr. No.	Antimicrobial combination	Frequency (%)
1	Cefotaxime + Metronidazole	95 (11.5)
2	Ceftriaxone + Metronidazole	36 (4.4)
3	Ciprofloxacin + Metronidazole	18 (2.2)
4	Amoxiclav + Metronidazole	14 (1.7)
5	Ciprofloxacin + Cefoperazone/Sulbactam	11 (1.3)
6	Ceftriaxone + Clarithromycin	10 (1.2)

## Discussion

Though problems associated with the “less than optimal” use of antimicrobials exist all over the world, the gravity of the problem is higher in the developing countries where infection rates are high but resources are very limited [22, 23]. In this study, the practices associated with antimicrobial use have been investigated at ten wards selected within a tertiary care hospital which may help the policy makers for process improvement.

### Hospital indicators

The presence of FL/EML and STGs in a healthcare facility represents its commitment to provide good quality patient-care and promote rational use of medicines [22]. The BVH has a formulary list containing 25 generics of antimicrobials, approved by the hospital administration and it is revised on annual basis. There are however no STGs for infectious diseases (Table 3). In the absence of STGs for infectious diseases, prescribers do not have a standard to follow and they can prescribe antimicrobials freely, making it difficult to measure whether antimicrobial prescribing is rational or not. This may lead to an adverse impact on equitable access to essential drugs, thus compromising the quality of patient-care [22].

Besides the availability of STGs, it is essential that the key antimicrobials should be available all the time at hospitals. At the BVH, 93.8% of a set of key antimicrobials were available on the day of the study (Additional file 1: Table S4). This value is comparable with a study from Ethiopia that reported the availability of key antimicrobials in stock as 90.1% [34]. The average number of days that a set of key antimicrobials is out of stock indicates the capacity a healthcare facility has for maintaining stock and determines the procurement and proper distribution procedures. The resulting value for *indicator 4* was 3.3 days per month for the 32 key antimicrobials (Additional file 1: Table S5). This value was lower than those reported by studies conducted in Afghanistan (8.7 days per month for 15 key antimicrobials) [35] and Ethiopia (15–45 days over a 12-months period) [34]. The unavailability of key antimicrobials may force prescribers to prescribe medicines outside the FL/EML. Patients may not be able to get the drug of choice for particular infectious diseases, or they may be forced to buy branded or expensive medicines, or they might not receive any treatment at all. This may lead to economic burden on patients and non-compliance issues, as well as increased risk of morbidity and mortality [22].

Due to excessive and improper use of antimicrobials, the cost imposed by this single class of drugs is rising. *Indicator 5* records the cost of antimicrobials and demonstrates it as percentage of total hospital medicines costs. Results of the current study showed that the annual budget allocated for all medicines was USD 6.58 million of which the expenditure on antimicrobials (annual bulk purchase data only) was 12.2% (Additional file 1: Table S6). The reason for this low percentage was that the data from multiple and local purchase orders were not readily available and the data presented here is from a one-time annual purchase record.

### Prescribing indicators

*Indicator 6* determines the extent of antimicrobial prescribing in a healthcare facility. In our study, the percentage of hospitalizations with antimicrobial(s) prescribed was 82.3% (Table 4). This value was lower than that reported by studies from Afghanistan (90%) [35] and Nepal (93%) [36], and higher than Ethiopia (79.8%) [34], Thailand (44%) [37], Bangladesh (25%) [38], Tanzania (35.4%) [39] and Brazil (28.8%) [40]. During hospitalization, patients may be prescribed more than one antimicrobial. This prescribing may be appropriate according to the condition of patients, but it may also be a result of prescribing which is not optimal such as duplication of medicines, inappropriate use of combination therapy, and/or frequent and unnecessary alterations in dosage regimens [22]. In this study, the average number of antimicrobials per hospitalization (*indicator 7*) was 1.4 (SD = 0.6), and the difference among wards was found to be statistically significant (*p* < 0.005) (Table 4). A study from Ethiopia reported a lower value (1.2) [34], while studies conducted in Afghanistan (1.7) [35] and Nepal (2.4) [36] reported somewhat higher values. The study findings regarding antimicrobial prescribing showed that antimicrobials were prescribed to the majority of patients admitted to various wards of the BVH, but the number of antimicrobials being prescribed was not huge [22]. *Indicator 10* determines the length of time antimicrobials are prescribed and the extent of antimicrobial exposure to patients while they are hospitalized. The usual duration of treatment with antimicrobials for most of the infectious diseases is 7–10 days, but some diseases may also require longer durations such as osteomyelitis and meningitis [22]. In the BVH, the average duration of prescribed antimicrobial treatment was 5.4 days (SD = 3.2), and the difference among wards was found to be statistically significant (*p* < 0.005) (Table 4). A study from Afghanistan also reported comparable results of 5 days [35].

The WHO strongly recommends prescribing of medicines by their INN or generic names. *Indicator 14* measures the percentage of antimicrobials prescribed by their INN or generic names which was 52.5% at BVH (Table 4). This value was much lower compared to studies conducted in Afghanistan (88%) [35] and Thailand (87%) [37]. Antimicrobials prescribed by brand names may increase the chance of medication errors, thus prolonging morbidity and mortality along with putting extra financial pressure on individual the patients and healthcare budgets as a whole [41].

Since antimicrobials account for greater than 30% of total hospital budgets [15], this poses huge financial implications when they are not appropriately prescribed. *Indicator 9* measures the average cost of antimicrobials prescribed to hospitalized patients [22]. The findings of the current study demonstrated that the average cost of antimicrobials prescribed per hospitalization was USD 5.4 (SD = 6.7), and the difference among wards was found to be statistically significant (*p* < 0.005) (Table 4). In case of public sector hospitals in Pakistan, the government is the only financing agency which has to bear the healthcare costs. Unfortunately, there are no compulsory health insurance schemes in Pakistan. Therefore, USD 5.4 per patient on this single class of drugs (antimicrobials) may pose a huge financial burden on the government as well as on patients belonging to the lower income class. This may also be a reason for the unavailability and “stock out” of essential medicines and antimicrobials in the public sector hospitals, which has also been reported in our study. Similar to our findings, the cost of antimicrobials prescribed per hospitalization was USD 6.7 in Nigeria [42] whereas it was much lower in India at only USD 1.3 [43].

In cases of cesarean section surgical procedures, antimicrobial prophylaxis is recommended to avoid infections and the recommended regimen is the administration of one dose within one hour of the surgical procedure [44]. *Indicators 11 and 12* focus on the patients receiving surgical antimicrobial prophylaxis for cesarean section procedures. The assessment is whether they receive the prophylactic antimicrobial treatment in accordance with the clinical guidelines or not, and how many doses they receive [22]. The findings of this study showed that out of 100 cesarean section cases, 54 patients were prescribed surgical antimicrobial prophylaxis and none of these received this in accordance with the STGs. The average number of doses of surgical antimicrobial prophylaxis prescribed for cesarean section procedures was 1.2 (SD = 0.4) (Table 5). Since the hospital did not have its own STGs for surgical antimicrobial prophylaxis, previously published guidelines [45] were used as a reference.

It is necessary to follow the STGs for proper treatment of pneumonia or any other infection requiring antibiotics [35]. If STGs are unavailable or not followed adequately, there is a high probability that prescribing will be less than optimal and that utilization of antimicrobials will be excessive. This is likely to lead to increased incidences of ADRs, hospitalizations, and financial burden. The adherence of prescribers to the hospital’s STGs depends on two factors; prescribing only those antimicrobials listed in STGs; and prescribing within the dosage guidelines outlined in the STGs [22]. *Indicator 13* measures the percentage of pneumonia patients who receive antimicrobials in accordance with the appropriate STG. The results of the current study show that of the 28 reported cases of pneumonia none received antimicrobials in accordance with the clinical guidelines (Table 4). The hospital did not have STGs for pneumonia, therefore, previously published guidelines [46] were used as a reference. A study from Afghanistan also reported similar results that none of the patients received treatment in accordance with treatment guidelines [35].

### Patient-care and supplemental indicators

The frequency of performing antimicrobial sensitivity tests (*indicator 17*) is significant in determining the level of prescriber’s adherence to the STGs and the hospital’s ability to deliver appropriate antimicrobial treatment. The results of the current study reveal that drug sensitivity testing was almost non-existent; only 2 (0.24%) prescription records had a report of drug sensitivity tests (Table 6). This value is comparable with the studies from China (0.5%) [47] and Afghanistan (0.0%) [35]. Studies from Nigeria (20.53%) [48] and Nepal (19.8%) [36] reported higher levels of culture sensitivity testing.

The rational use of antimicrobials demands that patients should not be retained in hospitals longer than what is recommended in the STGs, and that they should also receive all prescribed doses of antimicrobials. *Indicator 15* determines the duration of stay at hospital for inpatients as an index of treatment effectiveness. The results of the current study reveal that the average duration of hospital stay of patients was 6.4 (SD = 4.3) days, and the difference among the wards was found to be statistically significant (*p* < 0.005) (Table 6). In most of the infectious disease cases, this duration of stay is acceptable. The results of the current study regarding *indicator 16* showed that all the prescribed doses of antimicrobials were administered to the hospitalized patients (Table 6), which is a good indication of rational use of antimicrobials; at least from the viewpoint of medicines adherence.

In-order to promote the rational use of medicines it is mandatory to follow the WHO’s recommendations regarding defined daily doses of medicines (DDD). There were only three antimicrobials (0.25 g ceftriaxone, 0.5 g clarithromycin and 0.4 g metronidazole) for which the mean observed doses were identical to the WHO recommended DDDs. Some drugs were given in lower DDDs than those recommended, such as 0.25 g cefotaxime. On the contrary, other antimicrobials were usually administered at a dosage that exceeded the WHO-recommended DDDs. The most extreme example of this was ciprofloxacin 0.4 g. WHO-recommended DDDs for 0.4 g ciprofloxacin was 0.5 g; whereas, the mean administered DDD was more than 37 fold i.e., 18.6 g (Table 7). This is of significant concern and warrants intervention at the levels of hospital policy and individual prescriber.

### Prescribing patterns of antimicrobials

There is a literature indicating that the consumption of antimicrobial is higher in developing than developed countries [49]. A study reported that 35 to 60% of patients were prescribed antimicrobials and less than 20% were prescribed appropriately [50]. In this study, out of 823 (82.3%) prescriptions with antimicrobial(s) prescribed, 536 (65.1%) had one antimicrobial, 252 (30.6%) included two and 34 (4.1%) had three antimicrobials. These findings could be compared with a Jordanian study [51], which indicated that out of 85% prescriptions with antimicrobials, 88% prescriptions had one, 11% had two and 1% had three antimicrobials. According to our findings, ceftriaxone (39.6%), metronidazole (23.4%) and cefotaxime (23.1%) were the most commonly prescribed antimicrobials (Table 8). The possible reasons behind high prescribing rates of these antimicrobials may include better clinical outcomes and excessive stock, or this might be the result of excessive and effective marketing strategies of pharmaceutical companies. A study performed in Ethiopia revealed that the most frequently prescribed antimicrobials were penicillin G (28.4%), ceftriaxone (24.9%) and cloxacillin (12.84%) [34]. An Indian study revealed that the most commonly prescribed antimicrobials were levofloxacin (25.77%), metronidazole (14.77%) and ceftriaxone (12.71%). Generally, the majority of infectious cases are treated for period less than two weeks, but severe and complicated cases may demand multiple antimicrobials for prolonged durations [52–54]. These multiple antimicrobial treatments are usually expected to provide broad antimicrobial cover [55]. The results of the current study reveal that the most frequently prescribed antimicrobial combinations were cefotaxime with metronidazole (11.5%) and ceftriaxone with metronidazole (4.4%) (Table 9). Indications for using these antimicrobial combinations include synergism, empirical therapy for poly-microbial infections and prevention of AMR development [56]. Usually, synergistic effects of antimicrobial combinations are desired when there are high risks of therapeutic failure with individual antimicrobials or greater probability of developing resistant strains [57, 58]. Recent studies rebut this argument through reporting synergistic combinations that may enhance the development of resistant strains [59–61]. Other associated risks with the use of antimicrobial combinations include development of super infections, greater toxicity and increased financial burden [56].

There are several limitations to this study. First, as antimicrobial use was investigated in one hospital the findings of this study cannot be generalized for the whole of Pakistan. However, it is a fact that uniform healthcare policies are implemented in all the hospitals by the Pakistani government. As such clinical practices are assumed to be consistent with practices in other tertiary care hospitals in Pakistan. Second, different wards in the tertiary-care hospital have varying degrees of antimicrobial use. As such, to address this bias mean values for each of the selected wards were calculated separately. The reasons behind prescribing practices and particularly those factors that lead to less than optimal use of antimicrobials were not explored. Further studies should focus on these issues.

## Conclusions

This was a drug utilization study using WHO methodology, conducted to explore the antimicrobial utilization in ten selected wards of the BVH, Bahawalpur, Pakistan. The results of the current study highlight that antimicrobial prescribing and utilization patterns are less than optimal. This is in terms of STG availability and compliance, antimicrobial stock out days, percentage of antimicrobial prescribing and prescribing of antimicrobials by INN, average cost per patient, and antimicrobial sensitivity testing; where things could be improved.

There are significant implications of this present study for policy and practice. Pakistan is a nation with a developing health care system and pharmaceutical policy is in its infancy. This study provides the impetus to bring into line prescribing practices in BVH and to set higher level policy via the DTC and implement training that ensures the optimal use of antimicrobials. Based on the study findings, it is recommended that DTC should develop and implement STGs for infectious diseases at the BVH. Patient-specific microbiological diagnosis of infectious diseases is also recommended. Infectious diseases specialist pharmacists should be appointed as they can play critical role in process improvement by developing and implementing a surveillance system in the hospital. They can search and provide data about the most common strains of microbes in Bahawalpur; and provide the necessary training to prescribing staff on a continuous basis. Policy makers should develop interventions that aim to minimize the costs associated with antimicrobial use. Studies based on these indicators should be conducted in all developing countries to obtain baseline and uniform data that will help in establishing international collaborations for the development of targeted interventions to promote the rational use of antimicrobials and the containment of AMR.

## Additional files

Additional file 1: Table S1. Study inclusion/exclusion criteria. **Table S2.** WHO hospital indicators. **Table S3.** WHO prescribing, patient-care and supplemental indicators. **Table S4.** Availability of a set of key antimicrobials in the hospital stores on the day of study (*Indicator No. 3*). **Table S5.** Average number of days that a set of key antimicrobials is out of stock (*Indicator No. 4*). **Table S6.** Percentage of individual antimicrobial costs based on the total cost of antimicrobials (*Indicator No. 5*). **Table S7.** Prescribing patterns of single antimicrobials at the selected wards of the Bahawal Victoria Hospital. (DOCX 34 kb)

Additional file 2: Bonferroni correction. (DOCX 26 kb)

## Abbreviations

ADRs: Adverse drug reactions
AM: Antimicrobial
AMR: Antimicrobial resistance
ANOVA: Analysis of variance
API: Active pharmaceutical ingredient
ATC: Anatomical therapeutic chemical
BVH: Bahawal Victoria Hospital
CDU: Chest disease unit
CH: Clinical history
DTC: Drug and therapeutic committee
ENT: Ear, Nose, Throat
FL/EML: Formulary list/Essential medicines list
INN: International nonproprietary names
MDR: Multiple drug resistance
PKR: Pakistani Rupee
STGs: Standard treatment guidelines
USD: United States Dollar
WHO: World Health Organization
