# Supplementary material for: Investigation of antimicrobial use at a tertiary care hospital in Southern Punjab, Pakistan using WHO methodology
Source: Antimicrob Resist Infect Control. 2017 Apr 28;6:41. doi: 10.1186/s13756-017-0199-7 (PMC5410053; doi:10.1186/s13756-017-0199-7)
Supplement: Supplementary file 1 — Study inclusion/exclusion criteria. Table S2. WHO hospital indicators. Table S3. WHO prescribing, patient-care and supplemental indicators. Table S4. Availability of a set of key antimicrobials in the hospital stores on the day of study (Indicator No. 3). Table S5. Average number of days that a set of key antimicrobials is out of stock (Indicator No. 4). Table S6. Percentage of individual antimicrobial costs based on the total cost of antimicrobials (Indicator No. 5). Table S7. Prescribing patterns of single antimicrobials at the selected wards of the Bahawal Victoria Hospital. (DOCX 34 kb) [file 13756_2017_199_MOESM1_ESM.docx]

**Additional file 1**

**Table S1: Study inclusion/exclusion criteria**

| **Sr. No.** | **Indicator** | **Inclusion / exclusion criteria** |
| --- | --- | --- |
| 1 | All | Inpatient prescription records, written for the period of January–June 2016, were included in the study. |
| 2 | All | The sample was limited to the encounters comprising of acute and chronic illnesses, including a mixture of health conditions and age of the patients. |
| 3 | All | Discharge notes were not considered in this study. |
| 4 | All | Referral, tuberculosis, human immunodeficiency virus and vaccination cases were excluded from the study. |
| 5 | Indicator 5 | Only the annual bulk purchase costs of antimicrobials were included in the study because multiple and local purchase data was not readily available. |
| 6 | Indicator 10 | This indicator includes only the duration of treatment with antimicrobials and does not include prophylaxis antimicrobial treatments. |
| 7 | Indicators 11, 12 | The prescription records with pre-existing infections were excluded from the study. |
| 8 | Indicator 14 | Generic or international non-proprietary names (INN) were those, as identified in the WHO list of INN. |

**Table S2: WHO hospital indicators**

| **Sr. No.** | **Hospital Indicators** | **Data Source** | **WHO Forms** |
| --- | --- | --- | --- |
| 1 | Existence of STGs for infectious diseases | Hospital director’s office,  DTC,  Pharmacy | Form 1 |
| 2 | Existence of an approved hospital FL/EML | Hospital director’s office,  DTC,  Pharmacy | Form 1 |
| 3 | Availability of a set of key antimicrobials in the hospital stores on the day of the study | Hospital medical stores,  Hospital pharmacy | Form 7 |
| 4 | Average number of days that a set of key antimicrobials is out of stock | Hospital medical stores,  Hospital pharmacy | Form 7 |
| 5 | Expenditure on antimicrobials as a percentage of total hospital medicine costs | Hospital medical stores,  Hospital pharmacy | Form 4,5,6 |

STGs = Standard treatment guidelines; DTC **=** Drug and therapeutic committee; FL/EML = Formulary list/essential medicines list

**Table S3: WHO prescribing, patient-care and supplemental indicators**

| **Indicator No.** | **Indicators** | **Data Source** | **WHO Forms** |
| --- | --- | --- | --- |
| **Prescribing indicators** | | | |
| 6 | Percentage of hospitalizations with one or more antimicrobials prescribed | Medical records department | Form 2 |
| 7 | Average number of antimicrobials prescribed per hospitalization in which antimicrobials were prescribed | Medical records department | Form 2 |
| 8 | Percentage of antimicrobials prescribed consistent with the hospital formulary list | Hospital pharmacy,  Medical records department | Form 2 |
| 9 | Average cost of antimicrobials prescribed per hospitalization in which antimicrobials were prescribed | Inpatient records,  Hospital pharmacy,  Medical records department | Form 2,5 |
| 10 | Average duration of prescribed antimicrobial treatment | Medical records department | Form 2 |
| 11 | Percentage of patients who receive surgical antimicrobial prophylaxis for cesarean section in accordance with hospital guideline | Operating theater,  Medical records department | Form 3 |
| 12 | Average number of doses of surgical antimicrobial prophylaxis prescribed for cesarean section procedures | Operating theater,  Medical records department | Form 3 |
| 13 | Percentage of patients with pneumonia who are prescribed antimicrobials in accordance with standard treatment guidelines | Medical records | Form 2 |
| 14 | Percentage of antimicrobials prescribed by generic name | Medical records department | Form 2 |
| **Patient-care and supplemental indicators** | | | |
| 15 | Percentage of doses of prescribed antimicrobials actually administered | Medical records department | Form 2 |
| 16 | Average duration of hospital stay of patients who received antimicrobials | Medical records department | Form 2 |
| 17 | Number of antimicrobial drug sensitivity tests reported per hospital admission with curative antimicrobials prescribed | Clinical history or laboratory | Form 2 |

**Table S4: Availability of a set of key antimicrobials in the hospital stores on the day of study (*Indicator No. 3*)**

| **Sr. No.** | **Antimicrobials** | **Availability on the day of study (No=0, Yes=1)** |
| --- | --- | --- |
|  | Inj. Amikacin Sulphate 500mg | 1 |
|  | Tab. Amoxiclav 625mg | 1 |
|  | Inj. Amoxiclav 1.2g | 1 |
|  | Susp. Amoxiclav 312.5mg/5ml | 1 |
|  | Inj. Ampicillin 500mg | 1 |
|  | Inj. Cefepime 1g | 1 |
|  | Cap. Cefixime 400mg | 1 |
|  | Inj. Cefoperazone + Sulbactam (1g) | 1 |
|  | Inj. Cefotaxime 250mg | 1 |
|  | Inj. Cefotaxime 1g | 1 |
|  | Inj. Ceftriaxone 250mg | 1 |
|  | Inj. Ceftriaxone 1g | 1 |
|  | Cap. Cephradine 500mg | 1 |
|  | Inj. Cephradine 500mg | 0 |
|  | Chloramphenicol + Dexamethasone | 1 |
|  | Tab. Ciprofloxacin 500mg | 1 |
|  | Inf. Ciprofloxacin 200mg/100ml | 1 |
|  | Drops Ciprofloxacin 0.3% | 1 |
|  | Tab. Clarithromycin 250mg | 1 |
|  | Tab. Clarithromycin 500mg | 1 |
|  | Inj. Clarithromycin 500mg | 0 |
|  | Cap. Doxycycline 100mg | 1 |
|  | Inj. Gentamycin 80mg/2ml | 1 |
|  | Inj. Imipenem monohydrate + Cilastatin (1g) | 1 |
|  | Tab. Metronidazole 400mg | 1 |
|  | Susp. Metronidazole 200mg/5ml | 1 |
|  | Inf. Metronidazole 500mg/100ml | 1 |
|  | Tab. Moxifloxacin 400mg | 1 |
|  | Inf. Moxifloxacin 400mg/250ml | 1 |
|  | Polymyxin B. Sulphate + Bacitracin eye ointment | 1 |
|  | Tobramycin + Dexamethasone | 1 |
|  | Inj. Vancomycin 500mg | 1 |
| **Total** | | **30** |
| **Percentage** | | **93.8%** |

Inj. = Injection; Tab. = Tablet; Susp. = Suspension; Cap. = Capsule; Inf. = Infusion

**Table S5: Average number of days that a set of key antimicrobials is out of stock (*Indicator No. 4*)**

| **Sr. No.** | **Antimicrobial name**  **(Dosage form, strength)** | **Days out of stock (Year 2016)** | | | | | | |
| --- | --- | --- | --- | --- | --- | --- | --- | --- |
|  |  | **June** | **May** | **Apr** | **Mar** | **Feb** | **Jan** | **Total** |
|  | Inj. Amikacin Sulphate 500mg | 0 | 0 | 0 | 30 | 11 | 0 | 41 |
|  | Tab. Amoxiclav 625mg | 0 | 0 | 0 | 0 | 0 | 0 | 0 |
|  | Inj. Amoxiclav 1.2g | 0 | 0 | 5 | 30 | 30 | 0 | 65 |
|  | Susp. Amoxiclav 312.5mg/5ml | 0 | 0 | 0 | 0 | 0 | 0 | 0 |
|  | Inj. Ampicillin 500mg | 0 | 0 | 0 | 0 | 0 | 0 | 0 |
|  | Inj. Cefepime 1g | 0 | 0 | 11 | 30 | 30 | 7 | 78 |
|  | Cap. Cefixime 400mg | 0 | 0 | 0 | 0 | 0 | 0 | 0 |
|  | Inj. Cefoperazone + Sulbactam (1g) | 0 | 0 | 15 | 11 | 0 | 0 | 26 |
|  | Inj. Cefotaxime 250mg | 0 | 0 | 0 | 0 | 0 | 0 | 0 |
|  | Inj. Cefotaxime 1g | 0 | 0 | 0 | 0 | 0 | 0 | 0 |
|  | Inj. Ceftriaxone 250mg | 0 | 0 | 0 | 0 | 0 | 0 | 0 |
|  | Inj. Ceftriaxone 1g | 0 | 0 | 0 | 0 | 0 | 0 | 0 |
|  | Cap. Cephradine 500mg | 0 | 0 | 0 | 8 | 17 | 0 | 25 |
|  | Inj. Cephradine 500mg | 14 | 0 | 0 | 0 | 0 | 0 | 14 |
|  | Drops Chloramphenicol + Dexamethasone | 0 | 0 | 0 | 0 | 0 | 0 | 0 |
|  | Tab. Ciprofloxacin 500mg | 0 | 0 | 0 | 0 | 0 | 0 | 0 |
|  | Inf. Ciprofloxacin 200mg/100ml | 0 | 0 | 0 | 0 | 0 | 0 | 0 |
|  | Drops Ciprofloxacin 0.3% | 0 | 0 | 0 | 0 | 0 | 0 | 0 |
|  | Tab. Clarithromycin 250mg | 0 | 0 | 0 | 0 | 0 | 0 | 0 |
|  | Tab. Clarithromycin 500mg | 0 | 0 | 0 | 0 | 4 | 15 | 19 |
|  | Inj. Clarithromycin 500mg | 0 | 0 | 14 | 30 | 30 | 30 | 104 |
|  | Cap. Doxycycline 100mg | 0 | 0 | 5 | 30 | 27 | 0 | 62 |
|  | Inj. Gentamycin 80mg/2ml | 0 | 0 | 0 | 0 | 0 | 0 | 0 |
|  | Inj. Imipenem monohydrate + Cilastatin (1g) | 0 | 0 | 7 | 30 | 20 | 0 | 57 |
|  | Tab. Metronidazole 400mg | 0 | 0 | 0 | 0 | 0 | 0 | 0 |
|  | Susp. Metronidazole 200mg/5ml | 0 | 24 | 30 | 18 | 0 | 0 | 72 |
|  | Inf. Metronidazole 500mg/100ml | 0 | 0 | 0 | 0 | 0 | 0 | 0 |
|  | Tab. Moxifloxacin 400mg | 0 | 0 | 0 | 19 | 6 | 0 | 25 |
|  | Inf. Moxifloxacin 400mg/250ml | 0 | 0 | 0 | 0 | 0 | 0 | 0 |
|  | Polymyxin B. Sulphate +Zinc Bacitracin ointment | 0 | 0 | 0 | 0 | 0 | 0 | 0 |
|  | Drops Tobramycin + Dexamethasone | 0 | 0 | 0 | 0 | 0 | 0 | 0 |
|  | Inj. Vancomycin 500mg | 0 | 0 | 0 | 30 | 13 | 0 | 43 |
| **Total number of days that a set of key antimicrobials is out of stock** | | | | | | | | **631** |
| **Average number of days that a set of key antimicrobials is out of stock** | | | | | | | | **19.7± 29.5** |
| **Average number of days that a set of key antimicrobials is out of stock per month** | | | | | | | | **3.3^*^** |

*Calculated based on six months data

Inj. = Injection; Tab. = Tablet; Susp. = Suspension; Cap. = Capsule; Inf. = Infusion.

**Table S6: Percentage of individual antimicrobial costs based on the total cost of antimicrobials (*Indicator No. 5*)**

| **Sr. No.** | **Generics’ Names** | **Annual purchase cost^*^ (USD)** | **Percentage** | **Cumulative percentage** |
| --- | --- | --- | --- | --- |
|  | Amikacin | 6,012.60 | 0.75 | 0.75 |
|  | Amoxiclav | 127,021.38 | 15.79 | 16.54 |
|  | Ampicillin | 4,867.34 | 0.60 | 17.14 |
|  | Ampiclox | 26,975.57 | 3.35 | 20.49 |
|  | Benzyl Penicillin | 181.70 | 0.02 | 20.51 |
|  | Cefepime | 11,681.62 | 1.45 | 21.96 |
|  | Cefixime | 44,092.38 | 5.48 | 27.44 |
|  | Cefoperazone + Sulbactam | 3,998.85 | 0.50 | 27.94 |
|  | Cefotaxime Sodium | 49,158.24 | 6.11 | 34.05 |
|  | Ceftazidime | 7,062.42 | 0.88 | 34.93 |
|  | Ceftriaxone Sodium | 132,781.90 | 16.50 | 51.43 |
|  | Cephradine | 3,702.04 | 0.46 | 51.89 |
|  | Ciprofloxacin | 39,165.39 | 4.87 | 56.76 |
|  | Chloramphenicol + Dexamethasone | 8,933.00 | 1.11 | 57.87 |
|  | Clarithromycin | 15,079.21 | 1.87 | 59.74 |
|  | Doxycycline | 2,710.44 | 0.34 | 60.08 |
|  | Gentamycin | 607.94 | 0.08 | 60.16 |
|  | Imipenem monohydrate + Cilastatin Sodium | 115,229.53 | 14.32 | 74.48 |
|  | Metronidazole | 70,785.46 | 8.80 | 83.28 |
|  | Moxifloxacin | 33699.18 | 4.19 | 87.47 |
|  | Piperacillin Sodium + Tazobactam | 54,614.43 | 6.79 | 94.26 |
|  | Polymyxin B Sulphate + Zinc Bacitracin | 4,867.34 | 0.60 | 94.86 |
|  | Trimethoprim + Sulfamethoxazole | 1622.45 | 0.20 | 95.06 |
|  | Tobramycin + Dexamethasone | 3,292.61 | 0.41 | 95.47 |
|  | Vancomycin HCl | 36,505.06 | 4.54 | 100.0 |
| **Total** | | **804,648.08** | **100.0** | **------** |
| **Total budget of medicines** | | **6,585,226.19** | **------** | **------** |
| **Percentage of antimicrobials cost based on the total medicines budget** | | **12.2%** | **------** | **------** |

**^*^**Only annual bulk purchase data; USD = United States Dollars.

**Table S7: Prescribing patterns of single antimicrobials at the selected wards of the Bahawal Victoria Hospital**

| **Hospital Wards** | **Number of hospitalizations with one or more AM(s)** | **Prescribing pattern of AM(s)^*^** | | |
| --- | --- | --- | --- | --- |
|  |  | **First most frequently prescribed AM**  **(hospitalizations)** | **Second most frequently prescribed AM**  **(hospitalizations)** | **Third most frequently prescribed AM**  **(hospitalizations)** |
| 1. CDU | 91 | Ceftriaxone (50) | Clarithromycin (19) | Cefotaxime (11) |
| 2. ENT | 89 | Cefotaxime (62) | Ciprofloxacin (15) | Amoxiclav (12) |
| 3. Gynecology | 91 | Cefotaxime (91) | Metronidazole (91) | ------- |
| 4. Medical 1 | 89 | Ceftriaxone (63) | Augmentin (18) | Metronidazole (17) |
| 5. Medical 2 | 91 | Ceftriaxone (71) | Amoxiclav (15) | Metronidazole (15) |
| 6. Nephrology | 63 | Amoxiclav (26) | Cefotaxime (22) | Ciprofloxacin (14) |
| 7. Orthopedics | 65 | Ceftriaxone (51) | Cefoperazone/Sulbactam (15) | Cephradine (13) |
| 8. Surgical 4 | 95 | Ceftriaxone (65) | Metronidazole (37) | Amoxiclav (26) |
| 9. Skin | 63 | Amoxiclav (58) | Ciprofloxacin (9) | Metronidazole (8) |
| 10. Urology | 86 | Cefoperazone/Sulbactam (46) | Ciprofloxacin (33) | Ceftriaxone (14) |

AM = Antimicrobials; CDU = Chest disease unit; ENT = Ear Nose Throat
