# Supplementary material for: Investigation of antimicrobial use at a tertiary care hospital in Southern Punjab, Pakistan using WHO methodology
Source: Antimicrob Resist Infect Control. 2017 Apr 28;6:41. doi: 10.1186/s13756-017-0199-7 (PMC5410053; doi:10.1186/s13756-017-0199-7)
Supplement: Supplementary file 2 — Bonferroni correction. (DOCX 26 kb) [file 13756_2017_199_MOESM2_ESM.docx]

**Additional file 2**

**Bonferroni correction**

| **Ward** | **Ward** | **Number of antimicrobial per encounter**  **p-value** | **Duration of treatment per encounter**  **p-value** | **Cost of antimicrobial per encounter**  **p-value** |
| --- | --- | --- | --- | --- |
| **Chest Disease Unit** | Ear Nose Throat | N.S | N.S | N.S |
|  | Gynecology | **<0.0005** | N.S | **<0.0005** |
|  | Medical 1 | N.S | N.S | N.S |
|  | Medical 2 | N.S | N.S | N.S |
|  | Nephrology | N.S | N.S | N.S |
|  | Orthopedic | **0.004** | **<0.0005** | N.S |
|  | Surgical 4 | **0.027** | N.S | N.S |
|  | Skin | N.S | N.S | N.S |
|  | Urology | N.S | N.S | N.S |
| **Ear Nose Throat** | Chest Disease Unit | N.S | N.S | N.S |
|  | Gynecology | **<0.0005** | N.S | **<0.0005** |
|  | Medical 1 | N.S | N.S | N.S |
|  | Medical 2 | N.S | N.S | 0.006 |
|  | Nephrology | N.S | **0.003** | N.S |
|  | Orthopedic | N.S | **<0.0005** | N.S |
|  | Surgical 4 | N.S | N.S | 0.001 |
|  | Skin | N.S | N.S | N.S |
|  | Urology | N.S | N.S | N.S |
| **Gynecology** | Chest Disease Unit | **<0.0005** | N.S | **<0.0005** |
|  | Ear Nose Throat | **<0.0005** | N.S | **<0.0005** |
|  | Medical 1 | **<0.0005** | N.S | **0.003** |
|  | Medical 2 | **<0.0005** | N.S | N.S |
|  | Nephrology | **<0.0005** | N.S | **<0.0005** |
|  | Orthopedic | **<0.0005** | **<0.0005** | **<0.0005** |
|  | Surgical 4 | **<0.0005** | N.S | N.S |
|  | Skin | **<0.0005** | N.S | **<0.0005** |
|  | Urology | **<0.0005** | N.S | **<0.0005** |
| **Medical 1** | Chest Disease Unit | N.S | N.S | N.S |
|  | Ear Nose Throat | N.S | N.S | N.S |
|  | Gynecology | **<0.0005** | N.S | **0.003** |
|  | Medical 2 | N.S | N.S | N.S |
|  | Nephrology | N.S | **0.007** | N.S |
|  | Orthopedic | N.S | **<0.0005** | **0.029** |
|  | Surgical 4 | N.S | N.S | N.S |
|  | Skin | N.S | N.S | **0.001** |
|  | Urology | N.S | N.S | N.S |
| **Medical 2** | Chest Disease Unit | N.S | N.S | N.S |
|  | Ear Nose Throat | N.S | N.S | 0.006 |
|  | Gynecology | **<0.0005** | N.S | N.S |
|  | Medical 1 | N.S | N.S | N.S |
|  | Nephrology | N.S | N.S | N.S |
|  | Orthopedic | N.S | **<0.0005** | **<0.0005** |
|  | Surgical 4 | N.S | N.S | N.S |
|  | Skin | N.S | N.S | **<0.0005** |
|  | Urology | N.S | N.S | **0.019** |
| **Nephrology** | Chest Disease Unit | N.S | N.S | N.S |
|  | Ear Nose Throat | N.S | **0.003** | N.S |
|  | Gynecology | **<0.0005** | N.S | **<0.0005** |
|  | Medical 1 | N.S | **0.007** | N.S |
|  | Medical 2 | N.S | N.S | N.S |
|  | Orthopedic | N.S | **<0.0005** | N.S |
|  | Surgical 4 | N.S | N.S | N.S |
|  | Skin | N.S | N.S | **0.019** |
|  | Urology | N.S | N.S | N.S |
| **Orthopedic** | Chest Disease Unit | **0.004** | **<0.0005** | N.S |
|  | Ear Nose Throat | N.S | **<0.0005** | N.S |
|  | Gynecology | **<0.0005** | **<0.0005** | **<0.0005** |
|  | Medical 1 | N.S | **<0.0005** | **0.029** |
|  | Medical 2 | N.S | **<0.0005** | **<0.0005** |
|  | Nephrology | N.S | **<0.0005** | N.S |
|  | Surgical 4 | N.S | **<0.0005** | **<0.0005** |
|  | Skin | N.S | **<0.0005** | N.S |
|  | Urology | N.S | **<0.0005** | N.S |
| **Surgical 4** | Chest Disease Unit | **0.027** | N.S | N.S |
|  | Ear Nose Throat | N.S | N.S | **0.001** |
|  | Gynecology | **<0.0005** | N.S | N.S |
|  | Medical 1 | N.S | N.S | N.S |
|  | Medical 2 | N.S | N.S | N.S |
|  | Nephrology | N.S | N.S | N.S |
|  | Orthopedic | N.S | **<0.0005** | **<0.0005** |
|  | Skin | N.S | N.S | **<0.0005** |
|  | Urology | N.S | N.S | **0.003** |
| **Skin** | Chest Disease Unit | N.S | N.S | N.S |
|  | Ear Nose Throat | N.S | N.S | N.S |
|  | Gynecology | **<0.0005** | N.S | **<0.0005** |
|  | Medical 1 | N.S | N.S | **0.001** |
|  | Medical 2 | N.S | N.S | **<0.0005** |
|  | Nephrology | N.S | N.S | **0.019** |
|  | Orthopedic | N.S | **<0.0005** | N.S |
|  | Surgical 4 | N.S | N.S | **<0.0005** |
|  | Urology | N.S | N.S | N.S |
| **Urology** | Chest Disease Unit | N.S | N.S | N.S |
|  | Ear Nose Throat | N.S | N.S | N.S |
|  | Gynecology | **<0.0005** | N.S | **<0.0005** |
|  | Medical 1 | N.S | N.S | N.S |
|  | Medical 2 | N.S | N.S | **0.019** |
|  | Nephrology | N.S | N.S | N.S |
|  | Orthopedic | N.S | **<0.0005** | N.S |
|  | Surgical 4 | N.S | N.S | **0.003** |
|  | Skin | N.S | N.S | N.S |

Number of antimicrobial per encounter of Chest Disease Unit (mean = 1.19; SD = 0.419) is significantly different from Gynecology (mean = 2.00; SD = 0.000), Orthopedic (mean = 1.52; SD = 0.687) and Surgical 4 (mean = 1.45; SD = 0.665) ward. Number of antimicrobial per encounter of Ear Nose Throat (mean = 1.29; SD = 0.568) is significantly different from Gynecology (mean = 2.00; SD = 0.000) ward. Number of antimicrobial per encounter of Gynecology (mean = 2.00; SD = 0.000) ward is significantly different from Chest Disease Unit (mean = 1.19; SD = 0.419), Ear Nose Throat (mean = 1.29; SD = 0.568), Medical 1 (mean = 1.34; SD = 0.583), Medical 2 (mean = 1.30; SD = 0.483), Nephrology (mean = 1.30; SD = 0.463), Orthopedic (mean = 1.52; SD = 0.687), Surgical 4 (mean = 1.45; SD = 0.665), Skin (mean = 1.29; SD = 0.607) and Urology (mean = 1.30; SD = 0.510) ward. Number of antimicrobial per encounter of Medical 1 (mean = 1.34; SD = 0.583) is significantly different from Gynecology (mean = 2.00; SD = 0.000) ward. Number of antimicrobial per encounter of Nephrology (mean = 1.30; SD = 0.463) ward is significantly different from Gynecology (mean = 2.00; SD = 0.000) ward. Number of antimicrobial per encounter of Orthopedic (mean = 1.52; SD = 0.687) ward is significantly different from Chest Disease Unit (mean = 1.19; SD = 0.419) and Gynecology (mean = 2.00; SD = 0.000) ward. Number of antimicrobial per encounter of Surgical 4 (mean = 1.45; SD = 0.665) is significantly different from Chest Disease Unit (mean = 1.19; SD = 0.419) and Gynecology (mean = 2.00; SD = 0.000) ward. Number of antimicrobial per encounter of Skin (mean = 1.29; SD = 0.607) ward is significantly different from Gynecology (mean = 2.00; SD = 0.000) ward. Number of antimicrobial per encounter of Skin (mean = 1.29; SD = 0.607) ward is significantly different from Gynecology (mean = 2.00; SD = 0.000) ward. Number of antimicrobial per encounter of Urology ward is significantly different from Gynecology ward (mean = 2.00; SD = 0.000).

Duration of treatment per encounter of Chest Disease Unit (mean = 5.41; SD = 2.74) is significantly different from Orthopedic (mean = 2.58; SD = 1.32) ward. Duration of treatment per encounter of Ear Nose Throat (mean = 4.97; SD = 2.67) ward is significantly different from Nephrology (mean = 4.97; SD = 2.67) and Orthopedic (mean = 6.98; SD = 1.32) ward. Duration of treatment per encounter of Gynecology (mean = 4.97; SD = 2.67) ward is significantly different from Orthopedic (mean = 2.58; SD = 1.32) ward. Duration of treatment per encounter of Medical 1 (mean = 5.06; SD = 3.38) is significantly different from Nephrology (mean = 4.97; SD = 2.67) and Orthopedic (mean = 6.98; SD = 1.32) ward. Duration of treatment per encounter of Medical 2 (mean = 5.40; SD = 3.82) is significantly different from Orthopedic (mean = 2.58; SD = 1.32) ward. Duration of treatment per encounter of Nephrology (mean = 4.97; SD = 2.67) is significantly different from Ear Nose Throat (mean = 4.97; SD = 2.67), Medical 1 (mean = 5.06; SD = 3.38) and Orthopedic (mean = 2.58; SD = 1.32) ward. Duration of treatment per encounter of Orthopedic (mean = 2.58; SD = 1.32) ward is significantly different from Chest Disease Unit (mean = 5.41; SD = 2.74), Ear Nose Throat (mean = 4.97; SD = 2.67), Gynecology (mean = 4.97; SD = 2.67), Medical 1 (mean = 5.06; SD = 3.38), Medical 2 (mean = 5.40; SD = 3.82), Nephrology (mean = 4.97; SD = 2.67), Surgical 4 (mean = 5.83; SD = 3.26), Skin (mean = 5.50; SD = 2.74) and Urology (mean = 6.32; SD = 3.33) ward. Duration of treatment per encounter of Surgical 4 (mean = 5.83; SD = 3.26) unit is significantly different from Orthopedic (mean = 2.58; SD = 1.32) ward. Duration of treatment per encounter of Skin (mean = 5.50; SD = 2.74) ward is significantly different from Orthopedic (mean = 2.58; SD = 1.32) ward. Duration of treatment per encounter of Urology (mean = 6.32; SD = 3.33) ward is significantly different from Orthopedic (mean = 2.58; SD = 1.32) ward.

Cost of antimicrobial per encounter of Chest Disease Unit (mean = 4.16; SD = 8.34) is significantly different from Gynecology (mean = 8.65; SD = 6.03) ward. Cost of antimicrobial per encounter of Ear nose throat (mean = 2.80; SD = 2.36) ward is significantly different from Gynecology (mean = 8.65; SD = 6.03) ward. Cost of antimicrobial per encounter of Gynecology (mean = 8.65; SD = 6.03) ward is significantly different from Chest Disease Unit (mean = 4.16; SD = 8.34), Ear Nose Throat (mean = 2.80; SD = 2.36), Medical 1 (mean = 5.16; SD = 6.17), Nephrology (mean = 4.54; SD = 8.79), Orthopedic (mean = 2.20; SD = 3.24), Skin (mean = 1.49; SD = 3.16) and Urology (mean = 3.05; SD = 3.19) ward. Cost of antimicrobial per encounter of Medical 1 (mean = 5.16; SD = 6.17) is significantly different from Gynecology (mean = 8.65; SD = 6.03), Orthopedic (mean = 2.20; SD = 3.24) and Skin (mean = 1.49; SD = 3.16) ward. Cost of antimicrobial per encounter of Medical 2 (mean = 6.11; SD = 9.16) ward is significantly different from Orthopedic (mean = 2.20; SD = 3.24), Skin (mean = 1.49; SD = 3.16) and Urology (mean = 3.05; SD = 3.19) ward. Cost of antimicrobial per encounter of Nephrology (mean = 4.54; SD = 8.79) ward is significantly different from Gynecology (mean = 8.65; SD = 6.03) and Skin (mean = 1.49; SD = 3.16) ward. Cost of antimicrobial per encounter of Orthopedic (mean = 2.20; SD = 3.24) ward is significantly different from Gynecology (mean = 8.65; SD = 6.03), Medical 1(mean = 5.16; SD = 6.17), Medical 2 (mean = 6.11; SD = 9.16) and Surgical 4 (mean = 6.52; SD = 5.58) ward. Cost of antimicrobial per encounter of Surgical 4 (mean = 6.52; SD = 5.58) is significantly different from Ear Nose Throat (mean = 2.80; SD = 2.36), Orthopedic (mean = 2.20; SD = 3.24), Skin (mean = 1.49; SD = 3.16) and Urology (mean = 3.05; SD = 3.19) ward. Cost of antimicrobial per encounter of Skin (mean = 1.49; SD = 3.16) ward is significantly different from Gynecology (mean = 8.65; SD = 6.03), Medical 1 (mean = 5.16; SD = 6.17), Medical 2 (mean = 6.11; SD = 9.16), Nephrology (mean = 4.54; SD = 8.79) and Surgical 4 (mean = 6.52; SD = 5.58) ward. Cost of antimicrobial per encounter of Urology (mean = 3.05; SD = 3.19) ward is significantly different from Gynecology (mean = 8.65; SD = 6.03), Medical 2 (mean = 6.11; SD = 9.16) and Surgical 4 (mean = 6.52; SD = 5.58) ward.
